# The Droserasin 1 PSI: A Membrane-Interacting Antimicrobial Peptide from the Carnivorous Plant *Drosera capensis*

**DOI:** 10.3390/biom10071069

**Published:** 2020-07-17

**Authors:** Marc A. Sprague-Piercy, Jan C. Bierma, Marquise G. Crosby, Brooke P. Carpenter, Gemma R. Takahashi, Joana Paulino, Ivan Hung, Rongfu Zhang, John E. Kelly, Natalia Kozlyuk, Xi Chen, Carter T. Butts, Rachel W. Martin

**Affiliations:** 1 Department of Molecular Biology and Biochemistry, University of California, Irvine, CA 92697, USA; piercym@uci.edu (M.A.S.-P.); jbierma@lbl.gov (J.C.B.); mcrosby@uci.edu (M.G.C.); grtakaha@uci.edu (G.R.T.); 2 Department of Chemistry, University of California, Irvine, CA 92697, USA; paynebe@uci.edu (B.P.C.); jkelly1@ccny.cuny.edu (J.E.K.); natalia.kozlyuk@vanderbilt.edu (N.K.); cici2019@tamu.edu (X.C.); 3 National High Magnetic Field Laboratory, Tallahassee, FL 32310, USA; joana.paulino@gmail.com (J.P.); hung@magnet.fsu.edu (I.H.); zhangrongfu@gmail.com (R.Z.); 4 Departments of Sociology, Statistics, and Electrical Engineering and Computer Science, University of California, Irvine, CA 92697, USA; buttsc@uci.edu

**Keywords:** antimicrobial peptide, membrane protein, lipid-protein interactions, solid-state NMR, *Drosera capensis*, carnivorous plant

## Abstract

The Droserasins, aspartic proteases from the carnivorous plant *Drosera capensis*, contain a 100-residue plant-specific insert (PSI) that is post-translationally cleaved and independently acts as an antimicrobial peptide. PSIs are of interest not only for their inhibition of microbial growth, but also because they modify the size of lipid vesicles and strongly interact with biological membranes. PSIs may therefore be useful for modulating lipid systems in NMR studies of membrane proteins. Here we present the expression and biophysical characterization of the Droserasin 1 PSI (D1 PSI.) This peptide is monomeric in solution and maintains its primarily α-helical secondary structure over a wide range of temperatures and pH values, even under conditions where its three disulfide bonds are reduced. Vesicle fusion assays indicate that the D1 PSI strongly interacts with bacterial and fungal lipids at pH 5 and lower, consistent with the physiological pH of *D. capensis* mucilage. It binds lipids with a variety of head groups, highlighting its versatility as a potential stabilizer for lipid nanodiscs. Solid-state NMR spectra collected at a field strength of 36 T, using a unique series-connected hybrid magnet, indicate that the peptide is folded and strongly bound to the membrane. Molecular dynamics simulations indicate that the peptide is stable as either a monomer or a dimer in a lipid bilayer. Both the monomer and the dimer allow the passage of water through the membrane, albeit at different rates.

## 1. Introduction

Plant carnivory has been considered as part of defense mechanisms involving the jasmonate pathway [1]. In most cases, the leaves are modified for capturing prey, using a variety of mechanisms including pitfall traps, the unique snap trap mechanism of the Venus flytrap, and sticky flypaper traps, among others, and all of these plants must perform their digestion without any mechanical breakdown of prey tissues, such as mastication. In species with flypaper traps, such as the *Drosera*, digestion generally occurs in an exposed environment over a prolonged period of time, without the benefit of a pitcher or closed trap. These plants catch prey in the sticky polysaccharide mucilage of their leaf tentacles, which then wrap around the meal to increase contact with the digestive mucilage [2]. The digestion is thus exposed to variable physical conditions from changes in weather and has increased risk of opportunistic microbial growth from bacteria and fungi that compete for nutrients from the captured prey and potentially infect the plant tissue, causing disease [3]. In the Droseracaea, the evolution of carnivory was accompanied by the loss of many genes common to other plants, and the concomitant expansion of genes specifically related to carnivory [4]. For these reasons, carnivorous plants are a potential source of novel and useful antimicrobial peptides as well as digestive enzymes. Here, we focus on an antimicrobial peptide discovered from the genome of the Cape sundew, *Drosera capensis*. This plant is relatively easily cultivated and has been the target of genome sequencing [5] and enzyme discovery [6,7,8] efforts.

Analysis of putative digestive enzyme sequences from *D. capensis* revealed several aspartic proteases that contain a segment of about 100 residues called a plant specific insert (PSI). PSIs are mostly-helical domains that are often cleaved off during maturation and act as independent proteins [9]. Structurally, PSIs are categorized as saposin-like proteins, a protein family whose members have substantial sequence diversity but share a strongly conserved, compact tertiary fold, usually stabilized by three disulfide bonds [10]. Although the *S. tuberosum* PSI is monomeric in solution [11], it was crystallized as a dimer [9] and appears to oligomerize upon interaction with anionic membranes [12,13]. Membrane permeabilization can induce cytotoxicity and is a common antimicrobial defense. PSI-containing aspartic proteases from *D. capensis* may have been recruited for digestive function due to their ability to digest prey proteins as well as inhibit microbial growth during digestion. These functions may be separate or synergistic: PSIs in the digestive mucilage could simply serve to suppress the growth of pathogens, or may also function to make insect lipids more available for digestion.

In other plants, aspartic proteases containing PSIs are implicated in stress responses, senescence, and pathogen responses [14,15,16]. Their overall function involves interacting with lipid membranes in a variety of ways, including membrane localization, increasing the availability of membrane lipids for enzymatic processing, and permeabilizing membranes [17]. For example, in the cardoon, the PSI from the aspartic protease cardosin A plays a role in vacuole localization [18,19] and induces vesicle leakage below pH 5.5, whether or not it is attached to the parent aspartic protease [20]. Similarly, the well-studied PSI from the potato *Solanum tuberosum* disrupts lipid vesicles and bilayers [9] and has been shown to exhibit antimicrobial activity against plant and human pathogens [21]. Here we report the expression and characterization of recombinantly expressed Droserasin 1 PSI (D1 PSI) from *D. capensis*. Our results show that the D1 PSI forms a compact, stable structure and is indeed capable of disrupting and permeabilizing membranes and inhibiting microbial growth.

## 2. Materials and Methods

### 2.1. Sequence Alignment and Clustering

All sequences from the *Drosera capensis* genome [5] and the *Dionaea muscipula* transcriptome [22] that were previously annotated as coding for MEROPS A1 aspartic proteases using the MAKER-P (v2.31.8) pipeline [23] and a BLAST search against SwissProt (downloaded 8/30/15) and InterProScan [24] were examined for the presence of a PSI. Sequence alignment with the previously- characterized PSI from the *Arabidopsis thaliana* protease APA1_ARATH was used for quality control; proteins that did not contain a full-length PSI were not selected for modeling or further analysis. ClustalOmega was used to produce sequence alignments [25] for putative aspartic protease PSIs. The following settings were used: gap open penalty = 10.0, gap extension penalty = 0.05, hydrophilic residues = GPSNDQERK, and a BLOSUM weight matrix. This resulted in six complete PSIs from *D. capensis* and two from *D. muscipula*. Three previously-characterized aspartic proteases from other plants are also included as reference sequences [26,27,28]. The resulting PSI sequences were clustered by sequence similarity.

### 2.2. Structure Prediction

Structures for the PSIs were predicted using the Robetta [29] implementation of Rosetta [30]. This software uses a combination of comparative modeling and all-atom refinement based on a simplified forcefield, yielding five structures for each protein. For the PSIs, both open and closed conformations were observed in the models: we selected the lowest-energy representative of each type of structure for each PSI. The open conformation was observed as part of a dimer structure, which was also employed for subsequent modeling of the D1 PSI as described below. This process was performed for the *D. capensis* and *D. muscipula* PSIs, as well as a reference PSIs from *Arabidopsis thaliana*. The PDB files corresponding to the predicted structures for the PSIs reported in this manuscript are available in the Supplementary Information. Protein structure figures were generated using UCSF Chimera [31] and VMD [32].

### 2.3. Gene Construction, Expression, and Purification

Plasmids containing the DNA sequence of the *D. capensis* Droserasin 1 PSI (D1 PSI) genes were purchased from Integrated DNA Technologies (San Diego, CA, USA). Each gene was flanked by regions containing restriction sites for NcoI and XhoI and contained an N-terminal 6x His tag and a TEV protease cleavage sequence (ENLYFQG). The gene was amplified using oligonucleotide primers purchased from Integrated DNA Technologies (Coralville, IA, USA), and the resulting gene product was cloned into pET28a(+) vector (Novagen, Darmstadt, Germany). D1 PSI was overexpressed in SHuffle T7 *Escherichia coli* (New England Biolabs, Ipswich, MA, USA) using an autoinduction protocol [33] at 25 °C with the modification of adding 50 μM IPTG [34]. Cells were allowed to grow for at least 24 h, lysed via sonication, heated at 70 °C for 20 min to precipitate most *E. coli* proteins, and cell debris was removed by centrifugation. His-TEV-D1 PSI was purified on a Ni-charged Bio-Scale Mini Profinity IMAC Cartridges (Bio-Rad, Hercules, CA, USA) where bound protein was washed with low imidazole wash buffer followed by washing with 40% isopropanol wash buffer mixture and 40% DMSO wash buffer mixture to remove potentially bound lipids before elution. The His tag was removed with the use of a His-tagged TEV protease (produced in-house), over one week, (the time required to obtain a sufficient yield of His tag-free protein), followed by reapplication to Ni-charged Bio-Scale Mini Profinity IMAC Cartridges (Bio-Rad, Hercules, CA, USA) to remove His-tagged TEV protease and uncleaved His-TEV-D1 PSI. The final purification step consisted of applying the sample to a HiLoad 16/600 Superdex 75 pg gel filtration column (GE, Pittsburgh, PA, USA) in 10 mM phosphate buffer. D1 PSI was dialyzed into 10 mM phosphate, 0.05% NaN${}_{3}$, pH 6.9, for all experiments unless otherwise stated. The mass of the protein was confirmed by electrospray mass spectrometry. For ${}^{13}$C, ${}^{15}$N labeled protein samples, protein was expressed using an optimized high-cell-density IPTG-induction minimal media protocol [35]. Purification was performed in the same manner as for natural abundance protein.

### 2.4. Circular Dichroism

D1 PSI was diluted to 0.125 mg/mL with either 10 mM succinate, acetate, MES, or phosphate buffer at pH 4, 5, 6, and 7, respectively, for the collection of full circular dichroism (CD) spectra between 190 and 260 nm in a 10 mm quartz cuvette. Three accumulations were collected and no smoothing function was applied to the collected data. Additional spectra were taken under the same conditions but where the D1 PSI was reduced in 1 mM DTT before dilution into the final buffer. Measurements were taken on a J-810 spectropolarimeter (JASCO, Easton, MD, USA) equipped with a thermal controller.

### 2.5. Fluorescence Spectroscopy

UV fluorescence measurements were made on D1 PSI at a concentration of 0.2 mg/mL under the same buffer conditions as for CD spectra including unreduced and DTT-reduced protein for full emission spectra, with an excitation wavelength of 280 nm. Spectra were taken using a Cary Eclipse Fluorescence Spectrophotometer (Agilent, Santa Clara, CA, USA).

### 2.6. Characterization of Oligomeric State

0.5 mg/mL of purified D1-PSI with 6x His-tag were incubated in 50 mM ammonium formate for pH 3.5, 50 mM ammonium acetate for pH 4, 5, and 6 or 50 mM ammonium bicarbonate for pH 7 and 8. Intact protein samples were diluted 5-fold in water and then run on the SYNAPT G2-Si Mass Spectrometer (Waters, Milford, MA, USA) via direct injection in a 80/20% 0.1% formic acid/ACN running buffer. Samples were ionized by electrospray ionization with a capilary voltage of 2.7 kV and then separated by the T-Wave ion mobility collision cell (Waters, Milford, MA, USA). The ion series generated was then deconvoluted using the MaxEnt1 algorithm supplied by the MassLynx (Waters Milford, MA, USA) software package to calculate the mass of the proteins at each pH.

### 2.7. Antimicrobial Assay

Antimicrobial activity of D1 PSI was measured by growing *Pichia pastoris* in standard yeast extract, peptone, dextrose media (YPD) with the addition of 50 mM sodium phosphate and adjusted to pH 4, 5, 6, or 7, with or without 25 μM D1 PSI, peptide concentration was selected to match previously reported experiments [21]. Two replicates were performed at each pH value. Cells were grown in 15 mL culture tubes in a final culture volume of 1 mL. Cultures were inoculated with a pregrown culture, resulting in a starting optical density (OD) of 0.005 at 600 nm, and were allowed to grow for 48 h at 30 °C. After growth total cell yield was estimated by measuring the OD at 600 nm.

### 2.8. Vesicle Fusion Assay

Lipids used for experiments were extracted from either *E. coli* or *P. pastoris* using the Bligh and Dyer method [36]. The lipids were then dried under a stream of nitrogen gas and polar lipids were extracted using a cold acetone precipitation [37]. The *E. coli* or *P. pastoris* polar lipids were solubilized by repeated heating and cooling, between 42 °C and room temperature, in either 10 mM succinate pH 4, 10 mM acetate pH 5, 10 mM MES pH 6, or 10 mM phosphate pH 7. The solution was then run through a mini extruder equipped with a 100 nm polycarbonate membrane (Avanti Polar Lipids, Alabaster, AL, USA) to create large unilamellar vesicles (LUV) of approximately 100 nm in diameter. Vesicle size was monitored over time at the different pH with or without 50 μM D1 PSI using dynamic light scattering on a Zetasizer Nano ZS (Malvern Instruments, Malvern, UK).

### 2.9. Lipid Interaction Quantification

1-palmitoyl-2-oleoyl-glycero-3-phosphocholine (POPC), 1-palmitoyl-2-oleoyl-sn-glycero-3- phosphoethanolamine (POPE), 1-palmitoyl-2-oleoyl-sn-glycero-3-phospho-(1${}^{\prime }$-rac-glycerol) sodium salt (POPG), 1-palmitoyl-2-oleoyl-sn-glycero-3-phospho-L-serine sodium salt (POPS), and 1-palmitoyl-2-oleoyl-sn-glycero-3-phosphate sodium salt (POPA) were purchased from Avanti Polar Lipids (Alabaster, AL, USA). A solution of 10 mM sodium acetate pH 4.5 containing 75 μM of POPC, POPE, POPG, POPS, and POPA each was divided into two aliquots. One aliquot had His-tagged D1 PSI added to a final concentration of 0.04 mg/mL. Both solutions were sonicated for an hour at room temperature and applied to a Ni-charged Bio-Scale Mini Profinity IMAC Cartridges (Bio-Rad, Hercules, CA, USA). The aliquot lacking D1 PSI had the flow-through collected, while the elution was collected from the aliquot containing D1 PSI. Lipids were extracted from the flow-through and elution respectively using the Bligh and Dyer method [36]. A series of ten-fold dilutions were made for each and the resulting samples were run on a Xevo G2-XS QTof spectrometer (Waters, Milford, MA, USA) in positive mode, with an in-line ACUITY UPLC BEH C18 Column (Waters, Milford, MA, USA), where lipids were eluted with a water/isopropanol gradient containing ammonium formate. Only the sample concentrations within the linear range of ion intensities were used. Ion intensities were tabulated for each lipid species and normalized to the total ion count to estimate relative lipid proportions.

### 2.10. Solid-State NMR

Samples were prepared by evaporating away chloroform from 5 mg of POPC and POPA, respectively, under a stream of nitrogen gas. Lipids were sonicated in 0.5 mL methanol to redissolve lipids then 0.5 mL of water was added followed by 2 mg of ${}^{13}$C, ${}^{15}$N labeled D1 PSI dissolved in water. Protein lipid mixture was briefly sonicated, flash frozen and lyophilized. Lyophilized sample was then hydrated with 10 μL 10 mM acetate, 0.025% sodium azide, pH 4.5 buffer. The resulting sample was cycled between 42 °C and room temperature ten times. Spectra were taken at the National High Magnetic Field Lab (Tallahassee, FL, USA) using the 40 mm bore Series Connected Hybrid (SCH) magnet system, currently the highest-field NMR magnet [38]. Two-dimensional ${}^{13}$C-${}^{13}$C cross polarization [39] dipolar assisted rotational resonance [40] (CP DARR) spectra were obtained at 36 T, with a 2 mm CPMAS probe tuned to frequencies of ${}^{1}$H, ${}^{13}$C, ${}^{15}$N, with ferroshims, at a temperature of 10 °C, a 100 ms mixing time, and a MAS rate of 24.4 kHz [38].

### 2.11. Molecular Modeling and Analysis

Predicted monomer and dimer structures of D1 PSI as described above were modeled within lipid bilayers using atomistic molecular dynamics (MD) simulations. Open monomer and PSI dimer structures were first adjusted by adding disulfide bonds based on homology to the potato (*S. tuberosum*) PSI, with protonation states corrected for pH 5 using PROPKA3 [41]. For each respective structure, a POPC membrane patch was prepared using the VMD membrane plugin; for the monomer structure, a 100 Å square patch was used, with a 150 Å patch employed for the PSI dimer. The membrane patch was then centered within a TIP3P water box [42] of dimension 80 Å normal to the patch, the PSI was added within the membrane center, and 0.1M NaCl was added. (All structure preparation was performed using VMD [32].) The prepared system was then equilibrated as follows. Initially, with all atoms other than those of the lipid tails were held fixed, the system was minimized for 5000 iterations and simulated at 300 K for 0.5 ns. (All simulations were performed in NAMD [43] using the CHARMM36 force field [44] under periodic boundary conditions in an NpT ensemble at 1 atm pressure with Nosé-Hoover Langevin piston pressure control [45,46] and Langevin temperature control (damping coefficient 1/ps).) Water and lipid were then released (with the protein held fixed), and the system was then minimized for 1000 iterations and simulated for an additional 0.5 ns at 300 K; during this time, water was excluded from the lipid layer using the script of [47]. Following this, all atoms were released, and the system was equilibrated for 0.5 ns at 310 K. The final state of this trajectory was then used as the initial state for a production run of 100 ns at 310 K with frames collected at 10 ps intervals. This protocol was employed for both monomer and dimer structures.

Following simulation, water flow through PSI-induced channels was measured by counting transitions of water molecules through the membrane during each 10 ps observation window using a custom R [48] script using the bio3d library [49]. Underlying flow rates were estimated by Bayesian inference using an autoregressive latent rate model defined as

$$\lambda_{i}∼t_{\nu }\left(\rho \lambda_{i-1},\sigma \right)Y_{i}∼\mathrm{NegBin}\left(\lambda_{i},\phi \right),$$

where $\lambda_{i}$ is the log expected flow rate in window *i* (in units of counts per window), $Y_{i}$ is the number of water molecules transitioning in window *i*, ν and σ are the degree of freedom and scale parameters for drift in the latent rate function, ρ is an autocorrelation parameter, and ϕ is the overdispersion parameter of the negative binomial distribution. (Student’s *t* and negative binomial distributions were respectively employed because of the extremely bursty character of the flow process.) Weakly informative priors were used for all parameters, based on physically plausible limits for the processes in question; specifically, we take 1/ϕ∼HalfCauchy(0,5), σ∼HalfCauchy(0,5), ν∼Gamma(2,0.1), ρ∼Normal(0,1), and $\lambda_{1}∼\mathrm{Cauchy}\left(0,5\right)$. Posterior inference was performed using No-U-Turn Hamiltonian Monte Carlo sampling [50] for 2000 draws using four independent Markov chains; simulation was performed using the rstan package [51]. In addition to rate estimation, marginal distributions of transition counts over all observation windows were also obtained; these were compared to the distributions arising from Poisson distributions with equivalent expected values to assess overdispersion.

## 3. Results and Discussion

### 3.1. D. capensis and Related Plants Contain Several Aspartic Proteases with PSIs

**Figure 1:**
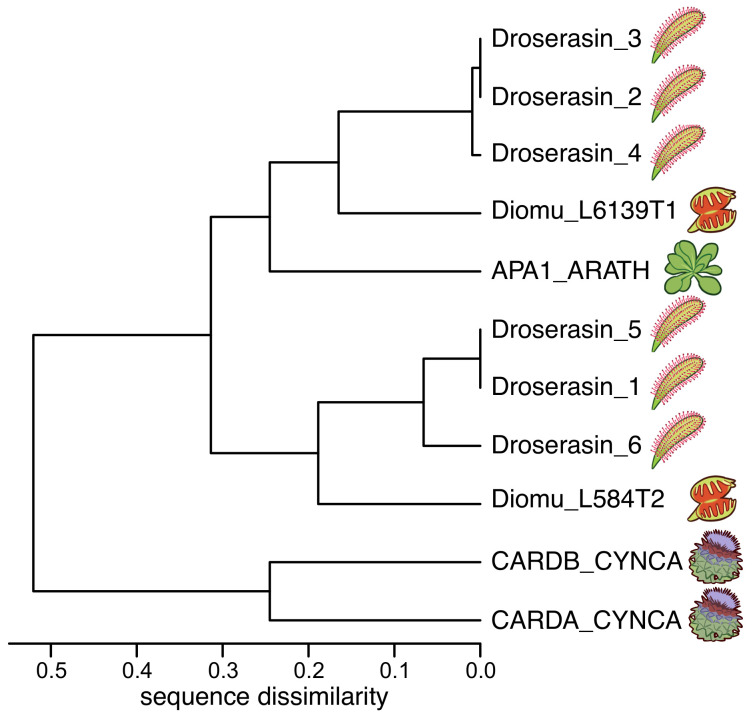
PSIs from *D. capensis* and *D. muscipula* clustered according to protein sequence similarity. Reference sequences from *Arabidopsis thaliana* and *Cynara cardunculus* are also included for comparison.

The genome of *D. capensis* contains at least six aspartic proteases with moderate sequence identity to mammalian pepsin (Droserasins 1–6) [5]. These proteases are classified as belonging to the MEROPS A1 class [52], which also includes pepsin and the nepethesins found in the digestive fluid of pitcher plants of the related genus *Nepenthes* [53,54]. The PSIs (without the catalytic domains) from *D. capensis* and *D. muscipula* were clustered by protein sequence similarity (Figure 1). Three well-characterized PSI sequences from other plants were also included for reference: *C. cardunculus* Cardosins A and B (Uniprot IDs CARDA_CYNCA and CARDB_CYNCA, respectively) and *A. thaliana* APA1 (Uniprot ID APA1_ARATH) [26,27,28]. Previous studies have shown that recombinantly expressed APA1_ARATH is maximally efficient at pH 5.3, and has a highly specific cleavage profile with respect to the insulin β-chain [55]. The full-length droserasins share important functional sequence features with the vacuolar protein APA1_ARATH and the cardosins, including the active site residues, the disulfide bonding pattern, and the PSI. Protein sequence alignments comparing sequences of the PSIs described here can be found in Supplementary Figure S1. All the PSI sequences share a relatively high degree of sequence identity, but the two from *C. cardunculus* are much more similar to each other than to the *D. capensis*, *D. muscipula*, and *A. thaliana* sequences. The latter group further clusters into two subtrees, with three representatives from *D. capensis* and one from *D. muscipula* in each group. The Droserasin 1 PSI (D1 PSI) was chosen for further modeling and experimental characterization as a representative of the group that is less related to the previously characterized APA1_ARATH.

**Figure 2:**
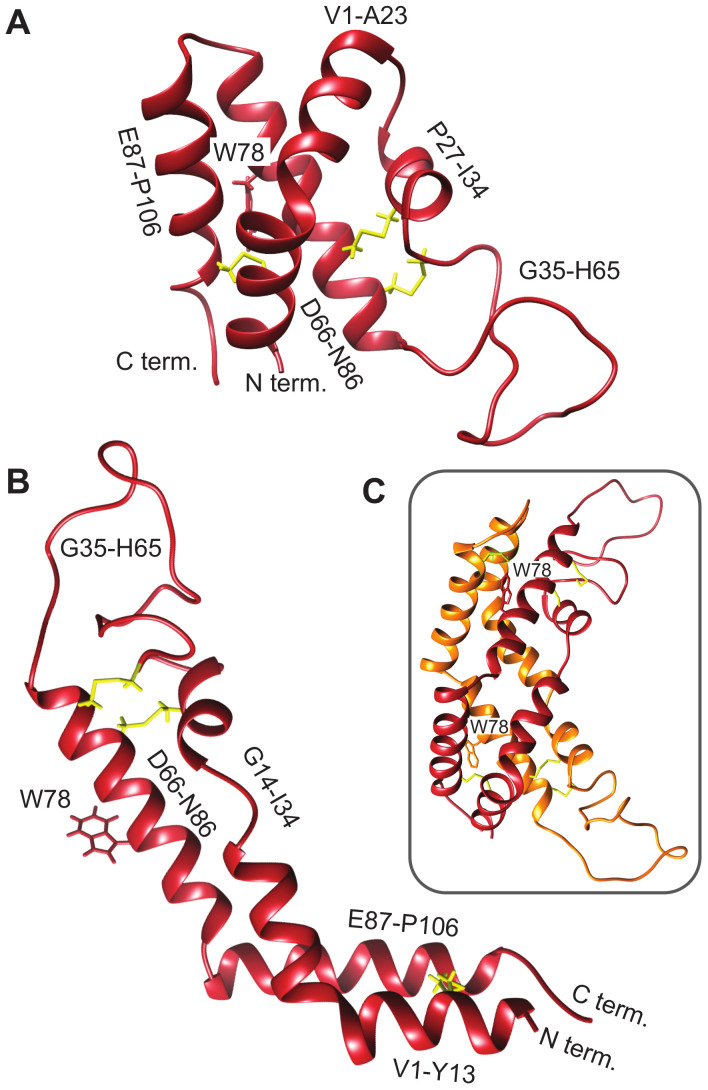
Comparative models of mature D1 PSI were predicted using the Robetta server [29,30]. (**A**) Closed conformation. (**B**) Open conformation. (**C**) Predicted dimer in solution.

Saposin-like proteins, including plant aspartic protease PSIs, are very stable, in part due to the presence of disulfide bonds that lock the tertiary structure into place [56]. This fold has two characteristic conformations, a compact closed form (Figure 2A) and an extended open form (Figure 2B), both of which can be adopted by human saposin C [57,58]. The crystal structure of the *S. tuberosum* PSI captures the open structure, observed as a domain-swapped dimer [9]. The corresponding model for the D1 PSI is shown in Figure 2C. The open conformation is proposed to be responsible for membrane-interacting activity due its increased exposure of hydrophobic residues compared to the closed conformation; in Section 3.7 we show via MD simulation that the open conformation of D1 PSI is indeed compatible with embedding in lipid bilayers. As we show, dimers formed from open-conformation monomers are also lipid-compatible, however; they have a distinct stabilization mechanism. The experiments and simulations that follow were performed in order to characterize the biophysical and membrane-interacting properties of the D1 PSI as a first step toward understanding its mode of antimicrobial activity.

### 3.2. D. capensis D1 PSI Is Highly Stable

In order to test the thermal stability of D1 PSI, experiments were performed with protein recombinantly expressed in *E. coli*. Typical yields of 20 mg/L of bacterial growth were achieved. Purification and characterization data are shown in Supplementary Figures S2 and S3. CD spectra were collected under different pH and temperature conditions. Regardless of the pH, the CD spectra collected at 20, 55 or 90 °C show very little variance (Supplementary Figure S4), indicating that at least the secondary structure of this protein is extremely thermostable. Furthermore, after pretreating the PSI with DTT to reduce the disulfide bonds, only a marginal reduction of signal is observed, indicating that the secondary structure is perturbed very little even after its three presumptive disulfide bonds have been reduced.

**Figure 3:**
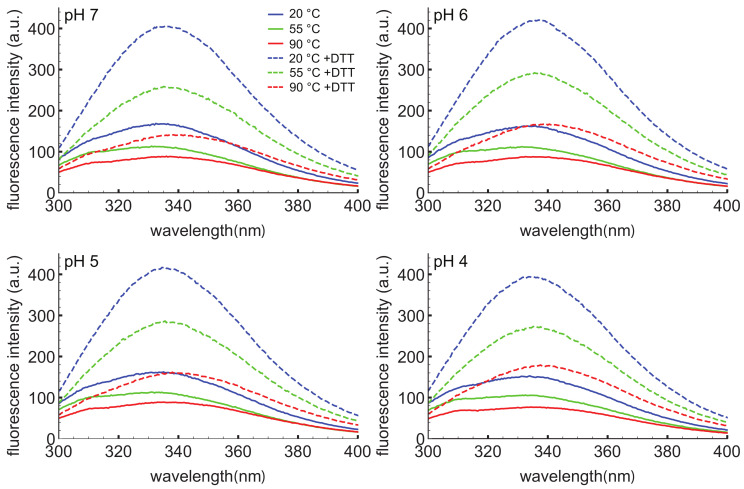
Intrinsic tryptophan fluorescence spectra of D1 PSI at pH 4, 5, 6, and 7. Spectra were collected at 20, 55 and 90 °C in the presence (dashed lines) or absence (solid lines) of DTT. The excitation wavelength was 280 nm. Under non-reducing conditions the emission maxima are at approximately 334 nm regardless of pH or temperature. Under reducing conditions the emission maxima for most of the spectra shift slightly to about 337 nm, indicating slightly more exposure of the single tryptophan. Reducing the protein also leads to an increase in the emission intensity, likely due to the reduction of a nearby disulfide bond that was quenching the tryptophan.

To further probe the response of the D1 PSI to pH, temperature, and reducing agent, intrinsic tryptophan fluorescence spectroscopy was employed (Figure 3). This technique can provide information on the local environment of the tryptophan as a more hydrophobic environment leads to a blue-shifted emission relative to a polar environment. The D1 PSI only has one tryptophan, allowing that particular position to be probed. Here, the intrinsic fluorescence was measured under the same conditions used for CD experiments. Under non-reducing conditions the wavelength of maximal emission is approximately 334 nm, regardless of temperature or pH, indicating that the single tryptophan (W78) is moderately exposed but insensitive to both pH and temperature. The emission intensity does decrease as a function of temperature, a known phenomenon in proteins [59]. When the D1 PSI has been reduced, there appears to be a slight shift in the emission maxima to approximately 337 nm, and more strikingly, the emission intensity increased relevant to the non-reduced PSI at equivalent temperature. A likely explanation could be that in the oxidized form, the tryptophan is next to a disulfide bond that is capable of quenching it, as in the closed conformation shown in Figure 2A but not the open monomer shown in Figure 2B, where the single Trp is solvent-exposed. Reducing the adjacent disulfide would release the quenching [60].

### 3.3. D1 PSI Is Monomeric in Solution over a Wide pH Range

There is experimental evidence that PSI of *Solanum tuberosum* forms dimers under low pH conditions [13], leading to the hypothesis that the activation of *D. capensis* D1 PSI requires dimerization at low pH values. To test this hypothesis, the presence of monomeric and dimeric D1 PSI was measured by intact protein mass spectrometry at a range of pH values, from 3 to 8 (Supplementary Figure S5). A dimeric form of D1 PSI would be expected to predominate at pH 3.5 and 4 but not at pH 7 and above if there is pH-dependent dimerization. For each pH value measured, D1 PSI was found to exist predominately in the monomeric form. Notably even at low pH values (pH 3.5 and 4) the monomeric form of the protein predominates. Also, the relative amounts of monomer to dimer do not change much as pH changes. Taken together we conclude that the oligomeric state of D1 PSI in aqueous solution is primarily monomeric, and is not strongly pH-dependent. This suggests that either the active form of D1 PSI is the monomeric state or that lipid bilayer interactions are required for dimerization.

### 3.4. D1 PSI Enables Vesicle Fusion at Acidic pH

**Figure 4:**
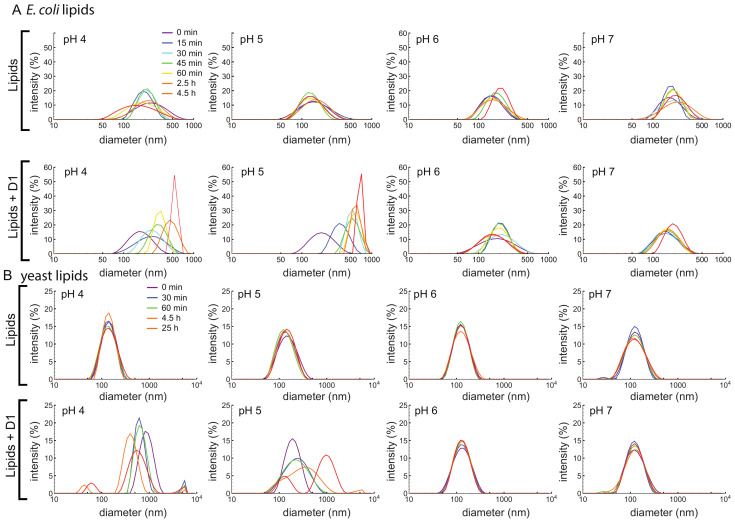
(**A**) LUVs of 100 nm were made using an *E. coli* polar lipid extract. LUVs were made in buffered solutions at pH 4, 5, 6, and 7. Vesicle size was monitored over time using DLS, either with (bottom) or without (top) D1 PSI. LUVs made with lipids alone are stable in size for all pH values, but upon addition of D1 PSI they fuse into larger vesicles at acidic pH. (**B**) LUVs of 100 nm were made using a yeast polar lipid extract. LUVs were made in buffered solutions at pH 4, 5, 6, and 7 and size was monitored using DLS over time, either without (top) or with (bottom) D1 PSI. Again, LUVs are stable in size for all pH values, but upon addition of D1 PSI they fuse into larger vesicles at acidic pH.

Based on previous studies in the literature, the simulations described in Section 3.7, and preliminary results consistent with inhibition of microbial growth at pH 5 (Supplementary Figure S6), we hypothesize that the PSI permeabilizes the membrane. We therefore used a vesicle fusion assay to test whether and how the D1 PSI interacts with membranes. Dynamic light scattering (DLS) was used to monitor increasing vesicle size due to fusion from membrane disruption. First, 100 nm large unilamellar vesicles (LUVs) were prepared using *E. coli* polar lipid extract. At all pH conditions tested, LUVs were stable in size over time as shown in Figure 4A. Upon addition of D1 PSI the distribution of LUV size begin to shift to larger sizes, but only at pH 4 and 5: the pH 6 and 7 samples do not change in size. DLS measurements of the D1 PSI alone were also recorded at each pH. For each sample, only one peak was present, corresponding to a size of about 2 nm, which was stable over time. When LUVs were made using yeast polar lipid extract instead, again the LUVs are stable over time at each pH (Figure 4B). When D1 PSI is added to the LUVs, only at pH 4 and 5 does the size distribution increase, similar to the results for the *E. coli* polar lipids. These findings are consistent with the antimicrobial assay in that D1 PSI interacts with membranes in a pH-dependent manner where it is only active at acidic pH. Another noteworthy observation is that with LUVs composed of *E. coli* polar lipids the rate of vesicle fusion was slow and gradual while in the case of the yeast polar lipids the change was very rapid at pH 4. Not only is the rate faster in this case, but for the pH 4 yeast polar lipid condition, smaller peaks appear at the latest time points. A potential hypothesis to explain the presence of these smaller particles after extended time is that it is possible that the D1 PSI is actually able to extract some of the lipids from the vesicle into small lipoprotein particles, consistent with the role of some saposins as surfactants.

### 3.5. D1 PSI Is Able to Interact with Lipids Having Diverse Head Groups

After showing that D1 PSI was able to interact with membrane lipids from a natural source that contains a variety of lipid species, we sought to gain insight into whether the D1 PSI preferentially binds specific polar lipids. The *S. tuberosum* PSI was found to interact with both anionic and neutral lipids, although its specific mode of membrane interaction does depend on the head groups, with more membrane disruption observed in negatively charged membranes [12]. Furthermore, the presence of cholesterol inhibits membrane fusion activity, making the PSI non-toxic to animal cells [11]. First, the affinity of D1 PSI for different phospholipid head groups was tested. Five phospholipid species containing different head groups, but the same acyl groups (a singly unsaturated 18-carbon acyl chain and saturated 16-carbon acyl chain), were tested for their ability to associate with the D1 PSI. A solution was prepared containing both the neutrally-charged POPC and POPE as well as negatively-charged POPG, POPS, and POPA. One aliquot of this lipid solution had His-tagged D1 PSI added and was mixed, allowing time for lipid-protein interactions to occur. Then the lipid-protein mixture was applied to Ni${}^{2+}$ resin where the PSI would bind, bringing with it any associated lipids, while unbound lipids were washed away.

**Figure 5:**
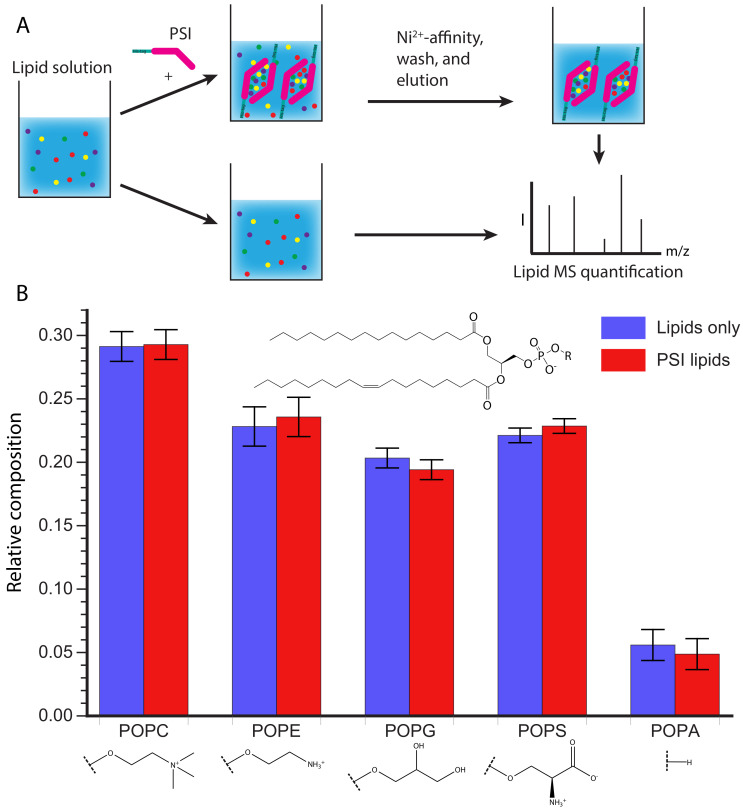
(**A**) Schematic representation of the experimental procedure. A lipid solution containing POPC, POPE, POPG, POPS, and POPA was prepared and split into two aliquots. One aliquot was treated with His-tagged D1 PSI and allowed to mix before purifying the His-tagged PSI and bound lipids from the unbound lipids. Lipids were then extracted from the D1 PSI and lipid solution and relative lipid species were quantified using mass spectrometry. (**B**) Results of lipid quantification. Blue represents lipids from the initial lipid mixture while red represents lipids that were bound to D1 PSI. The data indicate that there is no difference between the initial composition of lipids and the composition of lipids bound to the D1 PSI.

The PSI was eluted and any lipids that associated with it were quantified by MS and compared to the composition of the original lipid solution (Figure 5A). As shown in Figure 5B there is no significant difference in relative lipid composition between the original lipid solution and the lipids that co-purified with the PSI. However, it is possible the kinetics of association may be different depending on the lipid composition as hinted with the vesicle fusion assay (Figure 4). During membrane association the PSI must first interact with the head groups, before interacting with the acyl chains buried in the membrane. It is possible that specific head groups more strongly interact with the PSI, promoting initial association, followed by contact with the acyl chains, at which point PSI-acyl chain interactions predominate. The lack of sensitivity to the lipid head group makes the D1 PSI a promising candidate for making lipoprotein nanoparticles of different sizes for NMR studies of other membrane proteins, as previously demonstrated for saposin A [61,62,63].

### 3.6. Solid-State NMR Shows That D1 PSI Is Ordered and Strongly Bound to the Membrane

Solid-state NMR (ssNMR) is often used to solve the structures of membrane-associated proteins whose complexes with lipids are too large to tumble isotropically in solution. Because ssNMR makes use of magic angle spinning (MAS) to average out chemical shift anisotropy and dipolar coupling interactions it does not suffer the size limitations of solution-state NMR. This allows for the use of vesicles or bicelles as a membrane system to study membrane proteins [64]. Because of its similarity to saposins, the D1 PSI may be useful for stabilizing lipid nanodiscs, which are composed of a small section of lipid bilayer that is encircled by a membrane scaffold protein [65]. For ssNMR studies, ${}^{13}$C, ${}^{15}$N isotopically labeled D1 PSI was expressed, purified and mixed with a mixture of 1: 1 POPC and POPA at pH 4.5. The sample was sent to the National High Magnetic Field Laboratory in Tallahassee, FL to obtain spectra.

**Figure 6:**
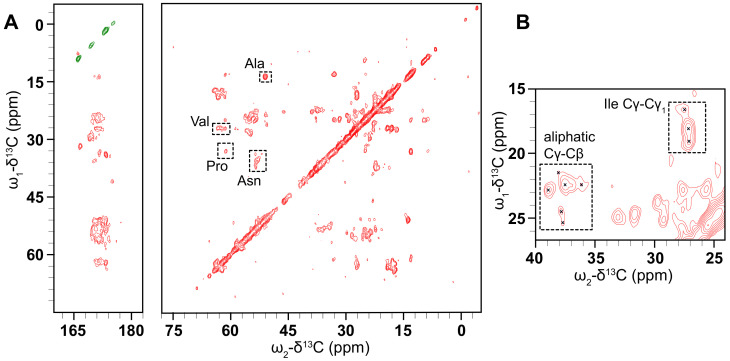
(**A**) CO (left) and aliphatic (right) regions of a ${}^{13}$C-${}^{13}$C CP DARR spectrum of D1 PSI in a 1:1 POPC, POPA membrane system was taken at 10 °C and spinning at 24.4 kHz. The spectrum displays well-resolved off-diagonal peaks of reasonable intensity indicating that the PSI is suitable for ssNMR structure determination. Tentative assignments are labeled by residue type. (**B**) The inset region shows some representative cross-peaks in the aliphatic region.

Our NMR investigations of this protein begin with 2D ${}^{13}$C-${}^{13}$C correlation spectra collected using the dipolar-assisted rotational resonance (DARR) experiment [40,66]. Early experiments using nano- or microcrystalline preparations of small, globular model proteins such as BPTI [67], ubiquitin [68], GB1 [69], and the α-spectrin SH3 domain [70] demonstrated that this type of homonuclear correlation can be used to provide partial assignments. In the usual procedure, well-resolved resonances with distinctive chemical shift values are identified, followed by mapping of spin systems via a “walk” among the proximal ${}^{13}$C atoms of the sidechain. Full assignments can then be obtained using further 2D, 3D and 4D experiments [71,72], followed by the measurement of through-space distance restraints (often using these same simple homonuclear correlations), and finally, structure determination [70,73,74]. This methodology is also fully applicable to membrane proteins [75,76,77], most readily in cases where the protein adopts a well-ordered conformation in the membrane. The ${}^{13}$C-${}^{13}$C CP DARR spectrum (Figure 6) shows that there are clearly-defined peaks that are reasonably well-dispersed, demonstrating that the PSI is not overly mobile and is not structurally heterogeneous in the sample so as to distribute the signal over multiple chemical shifts. Additional DARR spectra were collected with mixing times of 50 and 400 ms (Supplementary Figure S7). These spectra are similarly well-resolved, and comparing the three spectra shows the expected increase in the number of cross-peaks with increasing mixing time, as correlations between more distant spin pairs are observed.

These preliminary spectra indicate that this protein is amenable to structure determination by ssNMR. Based on common ${}^{13}$C chemical shifts we can tentatively assign some residues based on correlations between the Cα and Cβ carbons. For example, 1 Ala, 3 Val, 1 Pro, and 4 Asn residues were tentatively assigned. This suggests that a backbone walk is feasible for D1-PSI in a POPC and POPA membrane system. Carbon chemical shifts were compared to the average chemical shifts from all the proteins in the BioMagResBank [78], gathered from http://www.bmrb.wisc.edu/ref_info/statful.htm as published on 14 May 2020. 3D experiments will be needed for complete resonance assignments. Further sample preparation, such as optimization of lipid composition and protein-lipid ratios, is also needed to increase the signal of the sample and to explore how the D1 PSI responds to different lipids in the membrane system.

### 3.7. Molecular Modeling Suggests Potential Stability of Both Monomeric and Dimeric D1 PSI within Membranes, and Indicates That both Induce Permeability

**Figure 7:**
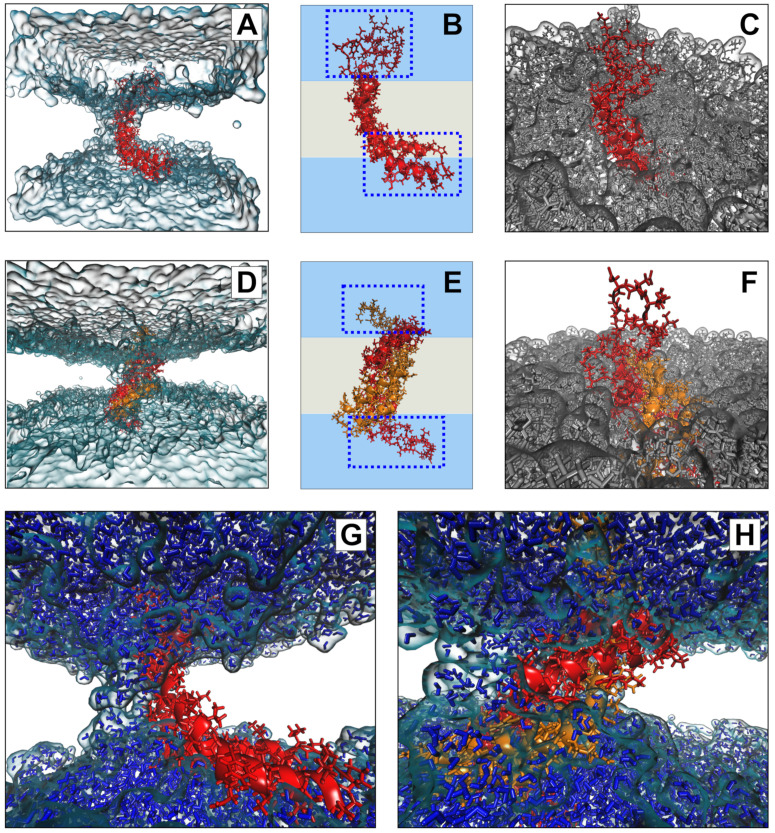
Conformations of monomeric (**A**–**C**) and dimeric (**D**–**F**) D1 PSI in POPC bilayer after 100ns. Both monomer and dimer assume stable but distinct conformations within the membrane (**A**,**D**), anchored by loop regions and (for the monomer) a helical “pontoon” as shown schematically in (**B**,**E**); blue regions indicate solvent, whereas grey regions indicate lipid. Loops extend substantially into solvent in both cases (**C**,**F**), while a depression forms in the surrounding bilayer. Bilayer depressions are linked to channels along the hydrophilic regions of the protein (**G**,**H**), through which water diffuses.

To further explore the potential interaction of the D1 PSI with membranes, in particular the question of whether the membrane-interacting form is likely to be monomeric or dimeric, we performed all-atom molecular dynamics (MD) simulations of the PSI within POPC bilayers. As both monomeric and dimeric conformations have been proposed to be biologically relevant for other PSIs, we examined both cases. After 100 ns (following initial stabilization and equilibration) at 310K, we see that both monomeric and dimeric forms retain stable-but quite distinct-conformations within the bilayer. Figure 7B provides a schematic depiction of the monomeric case. The PSI assumes the open “L” conformation, with the terminal helices V1–Y13 and E87–P106 forming a partially solvated “pontoon” that sits parallel to the lipid surface and the central helix pair (residues G14-I34 and D66-N86) spanning the bilayer at an angle of roughly 120 degrees with respect to the “pontoon”. On the opposite side, the protein is anchored by a mostly unstructured, hydrophilic loop region (G35-H65) that extends well into the solvent (see Figure 7C). This conformation appears to be stable, and indeed the “hinge” between the pontoon and the spanning helices would appear to allow the PSI to accommodate substantial deformation in membrane curvature or thickness without extensive conformational change.

In the case of the dimer, we observe a very different anchoring strategy. Figure 7E shows a schematic of the D1 PSI within the bilayer. In this case, the PSI forms a symmetric homodimer that anchors to both sides of the bilayer by respective copies of the hydrophilic anchor loop (G35-H65). Interestingly, the hydrophobic residues of the central helices that are lipid-exposed in the monomeric case serve here as the core of the homodimer, remaining stable despite burial in a hydrophobic environment. By contrast, the nominally lipid-exposed residues about the core (which form the “back” of the open “L” in the monomer, and the pontoon) are largely hydrophilic. This does not appear to destabilize the dimer; instead, these residues appear to facilitate the maintenance of a water channel, as described below. As with the monomeric case, the anchor loop is aggressively solvated, with a water layer extending several nanometers beyond the lipid head groups (Figure 7F). The highly flexible nature of the anchoring loops suggests a substantial entropic contribution to the free energy of the lipid-embedded dimer (more so than for the monomer), possibly allowing the dimer to remain anchored within the bilayer at higher temperatures or at higher ionic concentrations.

**Figure 8:**
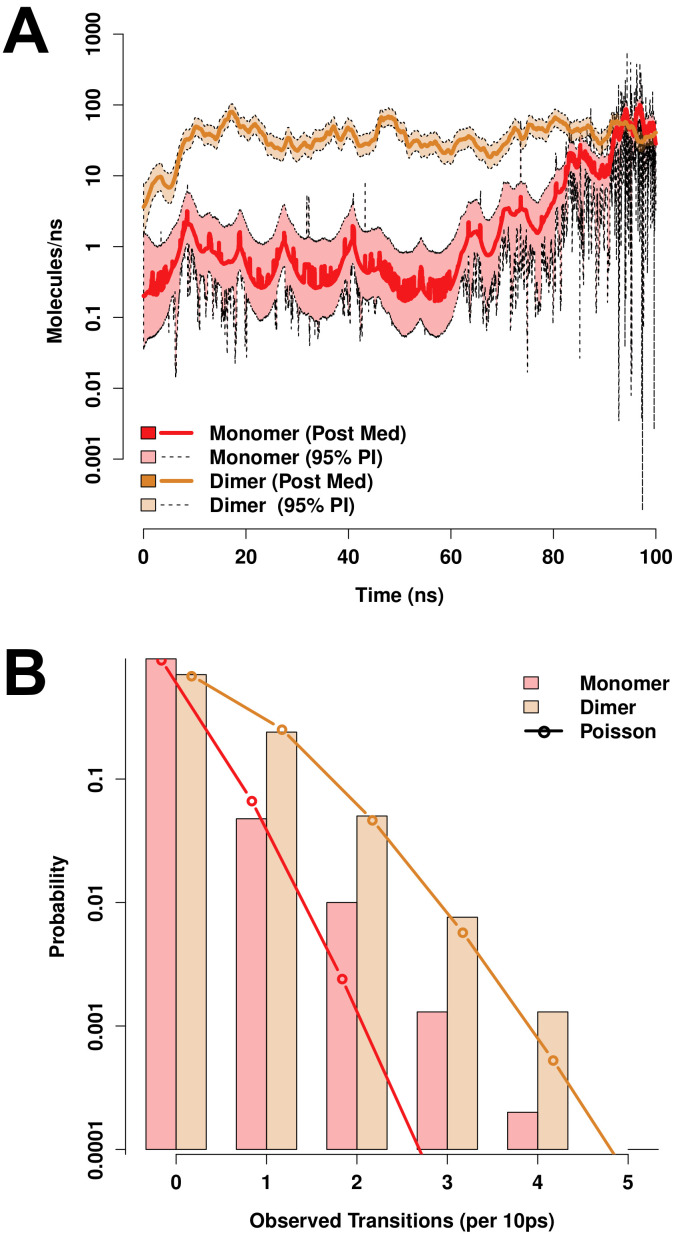
Both monomeric and dimeric D1 PSI conformations induce permeability in lipid bilayers, as revealed by MD trajectories. (**A**) Posterior median estimates (solid lines) and 95% posterior intervals (shaded areas) of trans-membrane water flow as a function of simulation time. Transport is extremely “bursty” in both conformations, with substantially larger fluctuations in mean rate for the PSI monomer trajectory. (**B**) Marginal distribution of transport events (cross-membrane water molecule transitions) per 10ns observation interval (bars). Observed distributions are heavier-tailed than Poisson distributions with equivalent expectation (lines), indicating high levels of “burstiness” even on short timescales.

Both monomeric and dimeric D1 PSI are observed to induce local changes in membrane conformation, creating “depressions” in the membrane surface (Figure 7C,F) that are linked to transmembrane water channels (Figure 7G,H). In the monomeric case, a single channel is formed that follows the hydrophilic residues on the “back” of the spanning helices, bridging to the hydrophilic residues on the sides and “bottom” of the pontoon (Figure 7G). For the dimer, the larger available hydrophilic surface area tends to produce multiple locally solvated regions (Figure 7H), supporting a broader channel structure. Examination of water transport across the membrane confirms that these apparent channels do indeed induce membrane permeability. Figure 8A shows posterior estimates of water transport rates (molecules/ns) across both simulated MD trajectories. As suggested by the broader channel structure, the PSI dimer supports a higher and somewhat more consistent mean flow rate (apx 37 molecules/ns) than the monomer (apx 7 molecules/ns), though the former is still somewhat “bursty” and irregular. Flow through the monomer channel is extremely irregular, with infrequent bursts and substantial changes in the overall flow rate over longer time scales (the former being evident from the roughness of the estimated rate function). Additional confirmation of the bursty nature of water flow in both cases can be seen by comparing the marginal distribution of observed transition events over short (10 ps) intervals to an equivalent Poisson process, as shown in Figure 8B. While the upper tail of the transition counts (i.e., numbers of water molecules observed to transition through the channel) is heavier than the Poisson in both cases, the departure is especially profound for the monomer trajectory. This may arise from the relative asymmetry of the channel structure (as shown in Figure 7G), which may create “pools” of water that can only pass when the monomer assumes specific conformations. Whether or not this is the case, the substantially higher mean flow rates for dimeric D1 PSI suggest that enhanced concentrations of PSI within membranes will produce greater than linear increases in membrane permeability, potentially contributing to toxicity in biological settings.

## 4. Conclusions

In conclusion, the D1 PSI is a mostly α-helical, highly thermostable peptide that maintains its secondary structure up to 90 °C, even under conditions where its three disulfide bonds are reduced. It causes vesicle fusion in lipid mixtures from both yeast and *E. coli*, but only at a pH ≤ 5, consistent with the physiological pH of *D. capensis* digestive mucilage. Mass spectrometry of bound lipids upon extraction from lipid mixtures indicate that the PSI interacts with a wide range of lipids, independent of the charge on the head groups. Solid-state NMR data indicate that it strongly interacts with the membrane in a bicelle mixture, consistent with its robust lipid interactions in vesicle fusion assays. Furthermore, these data suggest that the PSI adopts an ordered conformation when interacting with the membrane, making structural studies in the membrane-bound state feasible. A major question left to be resolved is the oligomeric state of the PSI in membranes. In aqueous solution, it is predominantly monomeric over a wide range of pH values from 3–8. However, MD simulations show that either the monomeric or dimeric state may interact with and permeabilize membranes. In both the monomeric an dimeric states, the PSI in its open conformation can span the membrane, inducing local changes in membrane curvature and allowing the passage of water molecules from one side to the other. Further experimental characterization is needed to elucidate the mechanism of membrane interaction, which may involve monomers, dimers, or even larger pore-forming complexes. 

## Supplementary Materials

The following are available online at https://www.mdpi.com/2218-273X/10/7/1069/s1, Figure S1: Sequence alignment for plant PSIs, Figure S2: SDS-PAGE gel showing expression and purification of D1-PSI, Figure S3: Electrospray mass spectrometry data of monomer, Figure S4: CD spectra at different pH values, Figure S5: Mass spec data at different pH values, Figure S6: *Pichia pastoris* cell yield data at different pH values, Figure S7: DARR spectra at different mixing times, Table S1: PSI models (.pdb format) available for download.

## Abbreviations

The following abbreviations are used in this manuscript:

D1	Droserasin 1
DMSO	dimethylsulfoxide
DTT	dithiothreitol
IPTG	Isopropyl β-D-1-thiogalactopyranoside
PSI	plant-specific insert
MES	2-(N-morpholino)ethanesulfonic acid
POPC	1-palmitoyl-2-oleoyl-glycero-3-phosphocholine
POPE	1-palmitoyl-2-oleoyl-sn-glycero-3- phosphoethanolamine
POPG	1-palmitoyl-2-oleoyl-sn-glycero-3-phospho-(1′-rac-glycerol)
POPS	1-palmitoyl-2-oleoyl-sn-glycero-3-phospho-L-serine
POPA	1-palmitoyl-2-oleoyl-sn-glycero-3-phosphate
LUV	large unilamellar vesicles
CD	circular dichroism
CP	cross-polarization
DARR	dipolar-assisted rotational resonance
MAS	magic angle spinning
MD	molecular dynamics
MS	mass spectrometry
NMR	nuclear magnetic resonance
